# Behavioral and neurocognitive phenotypes in Crigler-Najjar syndrome in Tunisia

**DOI:** 10.1192/j.eurpsy.2024.1219

**Published:** 2024-08-27

**Authors:** N. Bouayed Abdelmoula, R. Zekri, F. Bouaziz, K. Bensalem, M. Feki, A. Etteyeb, B. Abdelmoula

**Affiliations:** Genomics of Signalopathies at the service of Precision Medicine -LR23ES07, Medical University of Sfax, Sfax, Tunisia

## Abstract

**Introduction:**

Crigler-Najjar 1 (CN1) due to exon 3 mutations of the UGT1A1 gene is a not rare genetic disease in Tunisia with a founder effect. CN1 syndrome is very severe, and most of CN1 Tunisian patients die soon after birth, within a maximum of one year, due to kernicterus. Liver transplantation, which is the only available therapeutic method for CN1, remains unreachable.

**Objectives:**

The aim of this study was to report behavioral and neurocognitive phenotypes in CN1 patients who survived to school enrollment.

**Methods:**

We have selected all patients evaluated from 2004 to 2010, both clinically and molecularly, for a deficiency of bilirubin-UGT enzyme activity leading to a pathological elevation of unconjugated bilirubin with a suspicion of CN1 syndrome. Direct sequencing of targeted PCR amplification products was performed for molecular analysis of UGT1A1. Behavioral and mental features of patients were studied through our genetic counselling.

**Results:**

We identified 15 patients with the homozygous c.1070 A>G Tunisian mutation. Their age at diagnosis ranged from one week to 9 months for 13 patients. Six of them died within a month of molecular investigation. Only two boys were of school age, i.e. 6 and 9 years. The first had been hospitalized at 3 months year-old for a prolonged jaundice treated with phenobarbital and phototherapy. His psychomotor and neurological development was normal, with school attendance at the age of six. The second patient presented with an unexplored jaundice at the age of 3 days, which was later complicated by seizures and treated with phenobarbital. Despite neurological and motor sequelae associated to language impairments with slurred speech, he attended school at the age of six.

**Conclusions:**

The neurological and behavioral profile of CN1 patients depends on familial and medical management. Quick diagnosis, close follow up and early liver transplantation can improve prognosis.

**Disclosure of Interest:**

None Declared

